# Optimization of a Cell Counting Algorithm for Mobile Point-of-Care Testing Platforms

**DOI:** 10.3390/s140815244

**Published:** 2014-08-19

**Authors:** DaeHan Ahn, Nam Sung Kim, SangJun Moon, Taejoon Park, Sang Hyuk Son

**Affiliations:** 1 Real-Time Cyber-Physical System Laboratory, Daegu Gyeoungbuk Institute of Science and Technology (DGIST), Daegu 711-873, Korea; E-Mails: dahan@dgist.ac.kr (D.H.A.); son@dgist.ac.kr (S.H.S.); 2 Department of Electrical and Computer Engineering, University of Wisconsin-Madison, Madison, WI 53706, USA; E-Mail: nskim3@wisc.edu; 3 Cybernetics Laboratory, Daegu Gyeoungbuk Institute of Science and Technology (DGIST), Daegu 711-873, Korea; E-Mail: nanobiomems@dgist.ac.kr

**Keywords:** cell counting, point-of-care testing, normalized cross-correlation

## Abstract

In a point-of-care (POC) setting, it is critically important to reliably count the number of specific cells in a blood sample. Software-based cell counting, which is far faster than manual counting, while much cheaper than hardware-based counting, has emerged as an attractive solution potentially applicable to mobile POC testing. However, the existing software-based algorithm based on the normalized cross-correlation (NCC) method is too time- and, thus, energy-consuming to be deployed for battery-powered mobile POC testing platforms. In this paper, we identify inefficiencies in the NCC-based algorithm and propose two synergistic optimization techniques that can considerably reduce the runtime and, thus, energy consumption of the original algorithm with negligible impact on counting accuracy. We demonstrate that an Android™ smart phone running the optimized algorithm consumes 11.5× less runtime than the original algorithm.

## 1. Introduction

Human immunodeficiency virus (HIV) remains one of the most fatal and infectious viruses. Due to inadequate accesses to HIV prevention and treatment methods, HIV infection has rapidly spread in developing countries. Consequently, an estimated 35.3 million people worldwide were living with HIV in 2012 [1]. As one of diagnosis and care methods for such a disease, point-of-care (POC) testing can generate results quicker and, hence, can start the treatment much sooner than laboratory-based testing methods [2]. In particular, counting the number of specific cells in a blood sample is one of the most important tests in various biological and medical fields [3–6]; e.g., HIV testing exploits the fact that HIV-infected individuals have CD4^+^ T lymphocyte counts below 350 cells per microliter of blood [3].

There are manual and automatic methods for counting specific cells. The manual cell counting method is the most time-consuming and the most labor-intensive approach [7–9], which means it is not suitable for POC testing in a rapid, consistent and repeated manner [2]. Due to such limitations, many researchers have studied various automatic cell counting methods based on hardware approaches, such as electrical impedance measurement [10,11], optical inspection [12,13] or image processing [2,14,15]. The electrical impedance measurement method is a hardware-based technique that translates the change of impedance into a count of cells when particles pass through a small aperture. The optical inspection method is also a hardware-based technique that uses laser light to identify and count cells. However, both methods, which similarly exploit the physical properties of cells, are expensive.

Instead of relying on either manual inspection or costly hardware, software-based image processing approaches with inexpensive hardware, such as automated imaging cytometry and calculus-based cell counting [9,10], can be adopted as a cost-effective POC testing method. This is because such a cell counting method can be far faster and more accurate than the manual approach, while it is much cheaper than the aforementioned hardware approaches. For instance, a recently proposed approach [2] can count cells 5760× faster than the manual method at practically no equipment cost; it achieves this high performance by computing normalized cross-correlation (NCC) values between two images, *i.e.*, a blood sample image and a cell image in the library, to quantify the similarity between the two images. Such an approach for counting cells gives a few advantages over other methods, such as automated imaging cytometry; NCC computation is much faster and more accurate than other approaches, because it effectively utilizes the shape of a certain cell to locate and count cells.

**Figure 1.**
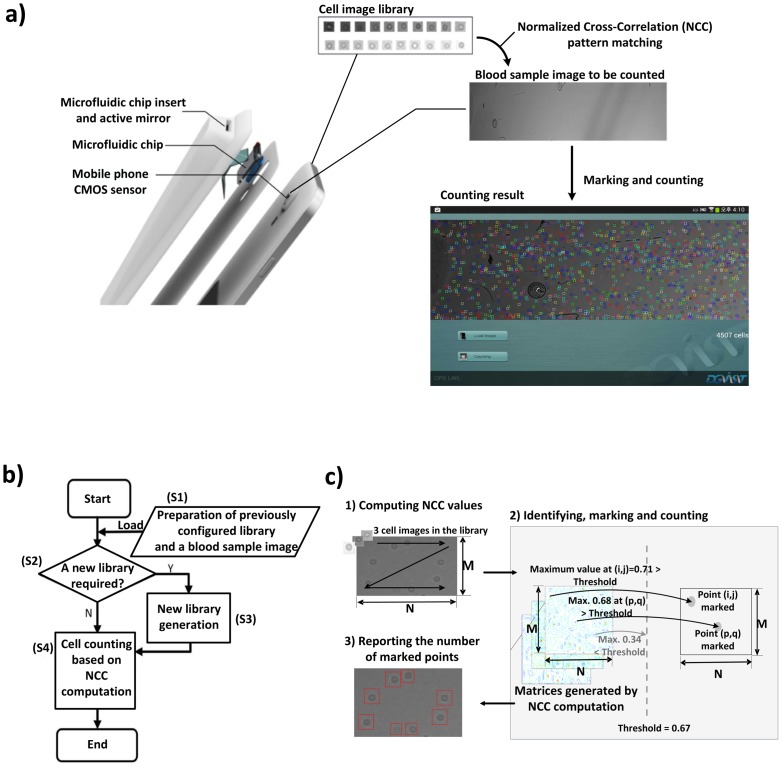
Schematic figure of an affordable point-of-care (POC) testing platform based on a commercially available smart phone, which can be integrated with a microfluidic system and an automated cell counting program. **(a)** The POC testing platform is composed of a smart phone CMOS sensor, a microfluidic chip and a chip cover (not yet available) containing an active mirror; it can simply be assembled with a commercially available smart phone by using a chip cover assay. The microfluidic chip is inserted into the cover assay and the CMOS sensor can take images with no optical lens. Note that actual cell images were captured using the apparatus in Figure 2. **(b)** The lensless image generated by an in-line holographic technique can be processed based on an Android™ smart phone, *i.e.*, a cell (shadow) image is counted by an automated cell counting program that consists of four steps: (S1) preparing a blood sample image captured by the imaging apparatus, as well as a pre-configured cell image library; (S2) checking whether or not a new cell image library is required for the captured image; (S3) generating a new cell image library if required; (S4) computing normalized cross-correlation (NCC) values for the entire image followed by identifying, marking and counting cells. **(c)** The actual counting process in (S4) first computes NCC values for the entire blood sample image by applying each and every cell image in the library in a zig-zag manner; then marking and counting point (*i, j*) as a cell if the maximum of NCC values for (*i, j*) exceeds a threshold, e.g., 0.67; and finally, reporting the number of marked points as counted cells.

The accessibility of POC tests has significantly expanded in both developed and developing countries [7,16]. However, hardware-based cell counting approaches have not been widely accepted yet, due to the high cost of procuring the necessary equipment and training the technicians for such sophisticated equipment [17]. On the other hand, mobile POC testing [17,18], which automates disease diagnosis using mobile devices such as smart phones and tablets, has emerged as an attractive alternative, as mobile devices become more affordable, portable and powerful. For example, Zhu *et al.* [18] presented a cost-effective imaging cytometry platform and custom-developed software for mobile POC testing. The schematic in Figure 1a shows how a commercially available smart phone running an automated cell counting algorithm can be used as an affordable POC testing device if integrated with a microfluidic platform in the form of a cover assay.

Despite such benefits, however, we note that mobile devices with a small form factor are still not suitable for the software-based methods. This is because such an algorithm often demands considerable computing capability to reliably deal with image anomalies, e.g., variations in background intensity, brightness and noise, while mobile devices with a small form factor have limited computing capability and battery capacity. For example, it takes more than 10 minutes for the algorithm running on an Android™ smart phone to count cells in a blood sample image. Therefore, it is crucial to optimize such a software-based approach for mobile devices with a small form factor to provide quick and uninterrupted services for an extended period of time.

In this paper, we propose two synergistic optimization techniques for the software-based algorithm based on NCC, such that it is suitable for Android™ smart phones after identifying the sources of its inefficiency. First, we observe that evaluating the NCC values for each point of a blood sample image with each and every cell image in the library is responsible for most of the runtime and energy consumption, both of which are proportional to the product of the number of cell images in the library and the number of points in the blood sample image. Second, we note that some cell images in the library are similar or duplicated because cell images are manually and randomly chosen. Meanwhile, these similar or duplicated cell images just increase the runtime and energy consumption without notably contributing to higher counting accuracy. Hereafter, we use runtime and energy consumption interchangeably, because they are proportional to each other. Third, a cell often spans across multiple points in a blood sample image, while evaluating NCC values for all of the points in the vicinity of a cell leads to detection of only a single cell. Thus, we note that evaluating NCC values for all of the points is highly redundant.

Motivated by these three observations, we propose the following two optimization techniques. First, we develop a technique to systematically remove duplicated or similar cell images from the library by evaluating the influence or loss of each cell image on the counting accuracy when removed. Second, we devise heuristic patterns that determine which points for which we skip NCC evaluations. Note that eliminating similar cell images in the library and/or skipping NCC evaluations for some points can decrease NCC values for points where cells are located. Meanwhile, these NCC values are compared against a threshold value to determine whether or not a cell exists in the point in a blood sample image. Consequently, the decreased NCC values for these points can incur some loss of counting accuracy. To compensate for the accuracy loss, we also propose to adjust the threshold value such that the degradation of counting accuracy is minimized.

We implement the optimized cell counting algorithm in an Android™smart phone and evaluate its runtime. The evaluation result demonstrates that the system adopting the proposed optimized algorithm outperforms the original algorithm, reducing the runtime by 11.5×. Accordingly, our work enables scalable solutions for realizing disposable low-cost POC testing platforms based on inexpensive mobile devices, thus offering quick blood tests for the diagnoses of diseases plaguing many developing countries.

## 2. Materials and Methods

### 2.1. Experimental Setup

**Figure 2.**
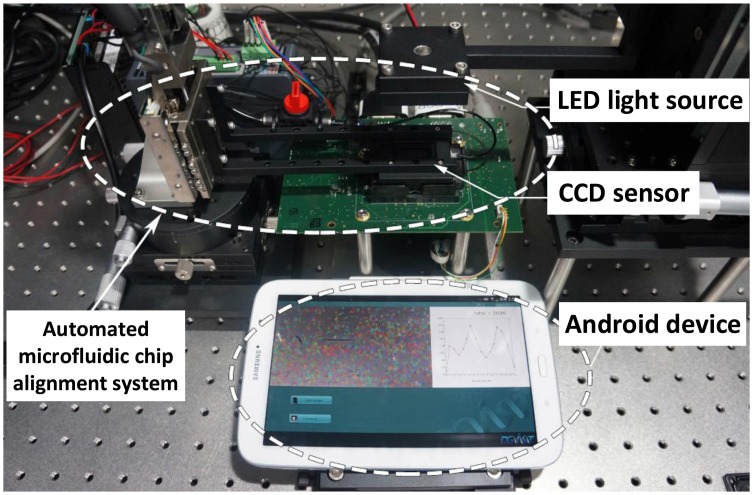
The cell imaging apparatus connected to an Android™ mobile device. The imaging apparatus is designed with an automated microfluidic chip alignment system, a syringe pump, a halogen lamp and a CCD imaging platform, while the Android™ device has a computing capability comparable to that of an Intel Atom processor, which is far slower than the typical processors for desktop platforms.

An Android™ smart phone is connected to an imaging apparatus shown in Figure 2 to capture 3334 × 445 blood sample images. The thus obtained images are used throughout the method development and validation. The specification of the smart phone is as follows: A 1.6-GHz quad-core CPU, a 533-MHz GPU, and 2-GB RAM running Android 4.1.2. Note that its computing capability is comparable to that of an Intel Atom processor, which is far slower than typical processors used for the desktop platform.

The imaging apparatus is designed for in-line holographic cell images on a microfluidic channel surface. The apparatus is designed with an automated microfluidic chip alignment system (cRIO, NI Korea, Korea), a syringe pump (Fusion 100 Touch, Revodix, Korea), a halogen lamp (FOK-100W, Micro-Lite, Korea) and a CCD imaging platform (Foveon F13 AVF-36A Development Kit, Alternative Vision Corporation, Tucson, AZ). Chip alignment with 10 *μ*m resolution is achieved with a precise manual *xy* stage (TPS-1, NAMIL Optical Co., Korea). To magnify in-line holographic cell images of targeted cells by 1.5 × magnification, the gap between the bottom of the microfluidic chip and the top surface of the CCD image sensor (Fx17-78-F13D-07v, Foveon Inc., USA) is determined as 1.1 mm. The halogen lamp, as a white light source, emits through a fifty-micron pin hole (#56282, Edmund Optics, Korea). The in-line holographic cell patterns are directly imaged on the CCD image sensor without a lens, where the CCD sensor features more than 14 million square pixels (7.8 *μ*m, pixel pitch) across the active sensor array area (24.9 mm, effective diagonal dimension) where the Foveon color CCD chip enables imaging with microscope cover slides (24 mm × 32 mm × 0.10 mm, Knittel Glaser, Germany).

As detailed in [2], NIH3T3 cells (mouse fibroblast cell line, CRL-1658) are cultured in Dulbecco's modified Eagle medium with high glucose (DMEM) containing 10% FBS, 100 U/mL penicillin, 100 g/mL streptomycin, and 2 mM L-glutamine. The NIH3T3 cells are maintained in the complete cell medium and grown at 37 °C in a humidified 5% CO_2_ environment.

After the cell culture process, a suspension of the NIH3T3 cells is injected (approximately 200 *μ*L total volume) into the microfluidic channel with a flow rate of 0.05 *μ*L/h. The controlled velocity of the cell containing fluid is generated by the automated syringe pump. The NIH3T3 cells refer to the targeted cells of the automatic cell counting sink and attach to the bottom of the microfluidic channel surface.

To obtain blood and cultured cell smears for imaging experiments, blood is first collected by pricking the finger with a needle. A small drop of blood sample is then dropped on the top of a slide glass. Tilting the cover slip at a 30° angle allows blood to spread across the edge. Thereafter, smoothly and quickly pulling the slide glass forward allows the drop of blood to spread even more thinly onto the slide. Next, the blood-stained slide glass is dried at room temperature for about 1 minute. Afterwards, the smeared blood film is dropped into 70% methanol and air-dried again before staining it by the Wright-Giemsa staining kit (Sigma-Aldrich Inc., St. Louis, MO, USA). The dried blood film is then placed into Coplin jars (5-PC glass staining jar, Citotest Labware Manufacturing Co., LTD., China), which contain approximately 50 mL Wright-Giemsa stain solution, for 30 seconds. Finally, the slide is removed from the stain and placed into de-ionized water or phosphate buffer solution (pH 7.2) for approximately 10 minutes and air-dried. This process allows the blood and cultured cell samples to be ready to be observed under the cell imaging apparatus.

### 2.2. Overview of Cell Counting

The cell counting algorithm based on NCC generally consists of four steps, as shown in Figure 1b: (S1) preparing a blood sample image captured by the imaging apparatus, as well as a pre-configured cell image library; (S2) checking whether or not a new cell image library is required for the captured image; (S3) generating a new cell image library if required; (S4) computing NCC values for the entire image followed by identifying, marking and counting cells. According to [2], if a generated library already exists (S2), there is no need to create another library (S3); otherwise, the cell library images must be manually predefined by marking cells located in random sections of the blood sample image (S3).

Computing NCC values allows us to identify cell locations in a blood sample image captured by any cell imaging apparatus [2,19]. Counting cells is essentially a process of searching for the similarity between the blood sample image and cell images from the library; since both of the images are from the same apparatus, it is capable of accurately locating all cells of a similar shape, thus reliably counting the number of cells. *R*(*i*, *j*), which denotes the NCC value at point (*i, j*) in a blood sample image, ranges from −1.0 to +1.0 and is calculated by:

(1) $$R(i,j)=\frac{\overset{L-1}{\underset{y=0}{\text{∑}}}\overset{K-1}{\underset{x=0}{\text{∑}}}\left(\omega (x,y)-\bar{\omega })(f(x+i,y+j)-\bar{f}(i,j)\right)}{\sqrt{\overset{L-1}{\underset{y=0}{\text{∑}}}\overset{K-1}{\underset{x=0}{\text{∑}}}{\left(\omega (x,y)-\bar{\omega }\right)}^{2}}\sqrt{\overset{L-1}{\underset{y=0}{\text{∑}}}\overset{K-1}{\underset{x=0}{\text{∑}}}{\left(f(x+i,y+j)-\bar{f}(i,j)\right)}^{2}}}$$

where 0 ≤ *i* ≤ *M* − *K* and 0 ≤ *j* ≤ *N* − *L*. *ω*(*x*, *y*), *f* (*x*, *y*), *ω̅* and *f̅* denote a cell image of *K* × *L* pixel points from the library, a blood sample image of *M* × *N* pixel points (e.g., 3, 334 × 445 pixel points in our implementation), a mean of *ω*(*x*, *y*) and a mean of a *K* × *L* sub-image of *f* starting at (*i*, *j*), respectively. The closer *R*(*i*, *j*) is to −1.0 or +1.0, the stronger the two images are correlated. On the contrary, the two images are highly irrelevant when the value is close to 0.

A threshold value is used to decide whether or not a cell exists at (*i*, *j*); if *R*(*i*, *j*) is higher than the threshold value, the cell counting algorithm marks point (*i*, *j*) as a cell. After computing the NCC values for the entire sample image with each and every cell image in the library, the algorithm counts the number of marked points and reports the number of counted cells as illustrated in Figure 1c. As the threshold value gets lower, the algorithm becomes more sensitive and perceives more cells, thus resulting in an increased count of cells. This indicates that determining an appropriate threshold value directly affects the accuracy of the algorithm. In [2], the threshold value was experimentally determined so as to minimize the difference between automated and manual counting results.

In the original NCC-based counting algorithm, however, we observe considerable inefficiency in Steps (S3) and (S4). First, a naive approach, which randomly chooses cell images to build the cell image library in (S3), often leads to more cell images than needed to achieve the desired accuracy, unnecessarily increasing the runtime proportional to the number of redundant cell images. Second, NCC values are evaluated for each and every point in (S4), while a cell in a blood sample image spans over multiple points. Thus, it is very likely to obtain high NCC values at multiple points in the vicinity of the cell. Since our objective is not to identify the exact center location of each cell, but to count the total number of cells, such multiple NCC evaluations for detecting a single cell are redundant. To avoid such inefficiency, we present two synergistic techniques optimizing the cell image library and approximating NCC evaluations.

### 2.3. Cell Library Optimization

Since the runtime is proportional to the number of cell images in the library, we propose a systematic method that can remove duplicated or similar images from the library without notably impacting the accuracy. This, in turn, reduces the number of cell images in the library and, thus, the runtime. In the original algorithm, 150 cell images were manually identified and randomly selected from various blood sample images to construct a cell image library. However, we observe that many similar cell images in the library do not significantly contribute to augmenting the counting accuracy, while increasing the runtime. In practice, it is inevitable to have multiple duplicated or similar images under such a random selection strategy. Therefore, we need a systematic method to eliminate those redundant cell images in the library.

**Figure 3.**
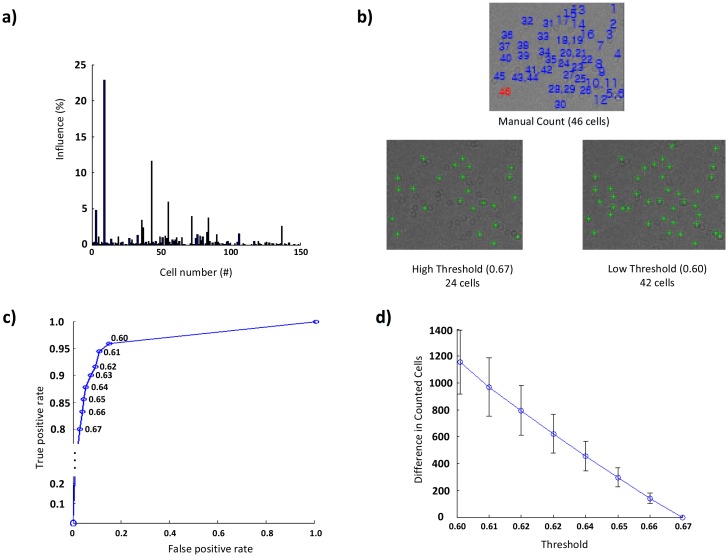
Cell library optimization to avoid inefficiency caused by random construction of the library. **(a)** The influence (%) indicates the proportion of the cell count of each cell image to the total counting result, which varies significantly from one cell image to another; some cell images cannot find any cells, because all of the cells found by these cell images are already counted by other duplicated and/or similar cell images. **(b)** This loss can further be compensated for by reducing the threshold value, e.g., 24 cells are marked under a high threshold value of 0.67, while 42 cells are identified after decreasing the threshold to 0.60, where the latter is close to the result of manual counting. **(c)** A receiver operating characteristic (ROC) curve plots a true positive rate (i.e., the ratio of the count of correct cell detection to the total cell count) *vs.* a false positive rate *(i.e.*, the ratio of the count of false cell detection to the total cell count) by varying the threshold value from 0.60 to 0.67. As shown in the ROC curve, the algorithm becomes less sensitive as the threshold value gets higher, thus resulting in the decreased count of cells. **(d)** The number of counted cells linearly depends on, or is inversely proportional to, the threshold value; decreasing the threshold value increases the number of counted cells. This relationship can be used to adjust the threshold value to counter the loss in counting accuracy after optimizing the library.

To further motivate our proposed approach, we evaluate the influence of each cell image regarding the accuracy by counting the number of cells discovered by a particular cell image in the library, as shown in Figure 3a, where the influence (%) indicates the proportion of the cell count of each cell image to the total counting result. In the library, some cell images cannot find any cells to be counted, because all of the cells, which are found by these cell images, are already counted by other duplicated and/or similar cell images. In the presence of similar cell images, the influence of some cell images in the library on counting accuracy is very small, because most of the cells identified by them are the same. Eliminating these duplicated or similar cell images incurs small loss in cell counting accuracy because cell images in the library can be slightly less similar to the removed cell images, leading to lower NCC values for some cells. However, we observe that such loss can be compensated for by reducing the threshold value. Based on these results, we present a joint optimization approach that: (i) removes images with the least losses from the library; and then (ii) adjusts the threshold to counter these losses.

### 2.4. NCC Approximation

The integral part of the cell counting algorithm consists of two steps, as illustrated in Figure 1c. First, it identifies the locations of cells by computing NCC values for each point with each and every cell image in the library; the points with their NCC values higher than the threshold value are identified as cells and are stored in (*M* − *K* + 1) × (*N* − *L* + 1) matrices, where *M* and *N* are the number of rows and columns in a blood sample image, while *K* and *L* indicate the size of a cell library image, as defined in Section 2.2. Second, all of the values in the matrix are read back to mark and count identified cells.

To reduce the runtime, we exploit the fact that a single cell usually appears as a cluster of points in the matrix, where a cluster has a maximum size of *m* × *n*, e.g., 3 × 3, as shown in Figure 3b. Since the cell counting algorithm may recognize a cell if the NCC value for at least one point of the cluster is higher than the threshold value, it is redundant to process each and every point on the blood sample image to evaluate NCC values. Hence, we may get an approximated counting result by skipping NCC evaluations for neighboring points as far as we can mark at least one point for a cell in the *M* × *N* matrix.

## 3. Results

In this section, we evaluate the accuracy and runtime of the cell counting algorithm, employing our proposed optimization techniques using an Android™ smart phone, and compare them against the original algorithm using the original library containing 150 randomly-selected cell images.

### 3.1. Cell Library Optimization

To build an optimized cell image library, we first evaluate the influence of each of the 150 cell images in the library used for our previous study for four different channels of the blood sample image. Figure 3a plots the influence of each cell image in the library on the counting result. We note that the influence of each cell image is almost the same for all channels, implying that our optimization technique is applicable to the entire blood sample image. Using our proposed optimization technique, we identify 38 duplicate images (with zero loss if removed) and 92 similar images (with <8.5% loss if removed).

**Figure 4.**
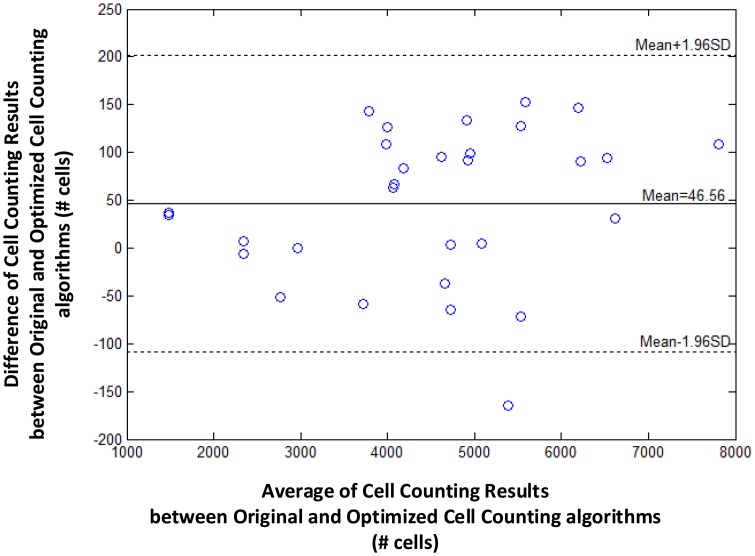
The Bland-Altman plot, evaluating the accuracy of the optimized cell counting algorithm in comparison with an original algorithm. The *x*-axis is the average of counting results of original and optimized algorithms, while the *y*-axis is the difference between them. It has a bias of 46.56 cells, and upper and lower limits of 201.72 and −108.60, respectively.

We further evaluate the loss in cell counting accuracy by enlarging the blood sample image. As shown in Figure 4, optimizing the cell image library by eliminating duplicated or similar cell images only incurs a small loss in counting accuracy regardless of the size of the blood sample image or the average count of cells. That is, the difference of cell counting results between original and optimized algorithms is sufficiently small (e.g., less than 200) even when the blood sample image is large enough (e.g., with 8000 cells).

The aggregated loss incurred by eliminating these images can be easily countered by reducing the threshold. Since the threshold value is closely coupled with the number of counted cells, it is important to reduce the threshold value, such that we can obtain the most compact library. We note that the threshold value of at least 0.6 indicates a strong correlation considering the correlation coefficient [20,21]. Therefore, eliminating most redundant cell images from the library and adjusting the threshold value from 0.67 (current value) to 0.60 allow us to obtain the optimized library with cell images that are essential to achieve a desired accuracy.

Figure 3b illustrates the sensitivity of cell counts to a threshold value; a cluster (of a size up to 3 × 3) indicates a cell. In the figure, 24 cells are marked under a high threshold value, while 42 cells are identified after decreasing the threshold. Based on this result, we conduct an experiment with six blood sample images to develop a model to compensate for the change in cell counts. We vary the threshold value from 0.67 to 0.60, where the latter indicates the lowest value to indicate a strong relationship [21], and compute the changes in the number of counted cells. As shown in Figures 3 c and d, the number of counted cells linearly depends on, or is inversely proportional to, the threshold value; decreasing the threshold value increases the number of counted cells. Let *C*(*T*) denote the number of counted cells, *α* the gradient, *β* the initial value and *T* the threshold value. Then, the following relationship holds:

(2) C(T)≈α·T+β

Equation (2) can be used to adjust the threshold value to counter the loss in counting accuracy after optimizing the library.

After the cell image library is optimized, the number of cell images in the original library is reduced from 150 to 20 with 8.5% accuracy loss on average. However, after we adjust the threshold value to 0.64 by using Equation (2) to compensate for the negative effect of fewer cell images in the library, we observe only negligible accuracy loss. Note that eliminating more cell images from the library yields noticeable accuracy loss that cannot be compensated for by simply adjusting the threshold value. With this optimized library containing only 13% (= 20/150) of cell images compared to the original library, we reduce the runtime by 87%.

### 3.2. NCC Approximation

The runtime reduction of NCC approximation can be computed as follows. If NCC values evaluated for every other point both horizontally and vertically, the runtime will be reduced to 25 of the original runtime. However, this method may slightly decrease the counting accuracy, because does not check all of the points, taking the risk of missing points associated with the center of cells that can give higher NCC values than other points. To minimize accuracy loss for such a case, we pres four heuristic patterns with the highest detection probability for a cell, as well as the shortest runtime, shown in Table 1.

As a metric to quantify the accuracy loss, a cell detection probability is derived as follows. Let *p_mark_* denote the probability that a point is marked as a cell because its NCC value exceeds the threshold. Furthermore, let *t* (= *m* × *n*) denote the total number of points in the cluster, e.g., set to nine for the maximum cluster size of 3 × 3, and *s* the number of skipped points in the cluster. Then, the detection probability, *P_k_*_|_*_org_*, which is the conditional probability that the algorithm with heuristic pattern k detects a cell within the cluster when the original algorithm has detected the cell, can be calculated by:

(3) $$P_{k|org}=\frac{1-{\left(1-p_{mark}\right)}^{s}}{1-{\left(1-p_{mark}\right)}^{t}}$$

Let us consider a critical region in which NCC values are close to the threshold since such a region dominates the accuracy loss caused by approximation. It is reasonable to assume *p_mark_* = 0.5 for points belonging to the critical region. Table 1 presents both *P_k|org_* calculated using this *p_mark_* and the rate of runtime reduction. The results show that employing Patterns 1, 2 and 3 reduces the runtime for evaluating NCC values by 50%∼75% at the expense of some accuracy loss (1 − *P_k_*_|_*_org_*, 1.4%∼6.1%). Specifically, Patterns 1 and 2 yield the highest detection probability (97.1%∼98.6%) and maintain the same amount of runtime reduction (50%), while Pattern 3 achieves a further reduction in runtime (75%) compared to Patterns 1 and 2, but its detection probability becomes lower (93.9%). Pattern 4 has the poorest detection probability (50.1%) and, hence, may not meet the accuracy requirement.

**Figure 5.**
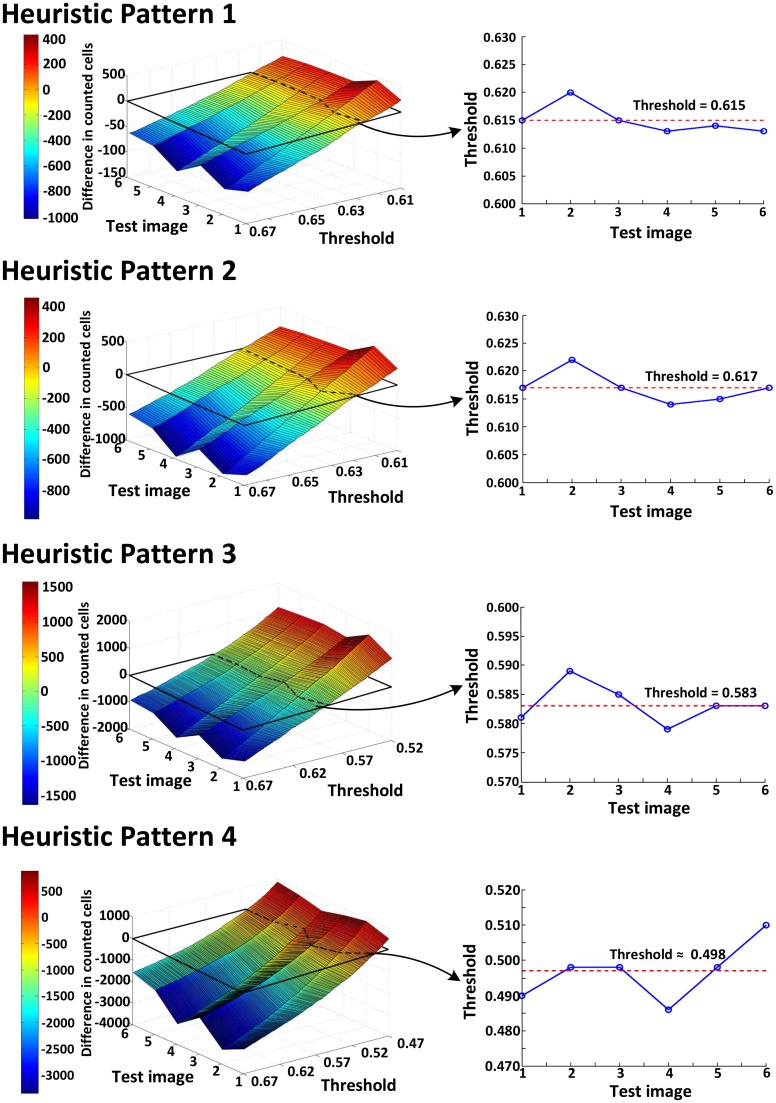
The difference in counted cells between the original algorithm and the algorithm with NCC approximation (heuristic Patterns 1∼4). The closer the difference is to zero, the more accurate the algorithm with NCC approximation gets. Since all four heuristic patterns for NCC approximation cross zero, it is possible to find the best threshold values that can practically eliminate the accuracy loss, which are 0.615, 0.617, 0.583 and 0.498 for heuristic Patterns 1, 2, 3 and 4, respectively. The heuristic patterns 1 and 2 satisfy the accuracy requirement (*i.e.*, zero loss) for the range of threshold (>0.6) and reduces the runtime required for NCC by 50%.

To compensate for the accuracy loss due to NCC approximation, we also adjust the threshold value using Equation (2), as we do for optimizing the cell image library. First, we compare the accuracy of the original algorithm with that of the algorithm with NCC approximation, both of which use the optimized cell library. Figure 5 plots the difference in counted cells between the two approaches; the closer the value is to zero, the more accurate the algorithm with NCC approximation gets. Since all four heuristic patterns for NCC approximation cross zero, it is possible to find the best threshold values that can practically eliminate the accuracy loss.

Figure 5 also presents the threshold values using four heuristic patterns for the same counting accuracy as the original algorithm. Clearly, the more points the algorithm skips, the shorter the runtime it achieves, as illustrated in Table 1. However, we need to decrease the threshold value to compensate for the increased counting loss. Our analysis shows that the heuristic Patterns 1 and 2 satisfy the accuracy requirement for the range of threshold (>0.6). With these heuristic patterns, we reduce the runtime required for NCC by 50%.

### 3.3. Measurement Using an Android™Device

We run both the original and optimized cell counting algorithms on an Android™ smart phone connected to the imaging apparatus shown in Figure 2 to evaluate the effectiveness of our proposed optimization techniques for 3334 × 445 blood sample images.

**Figure 6.**
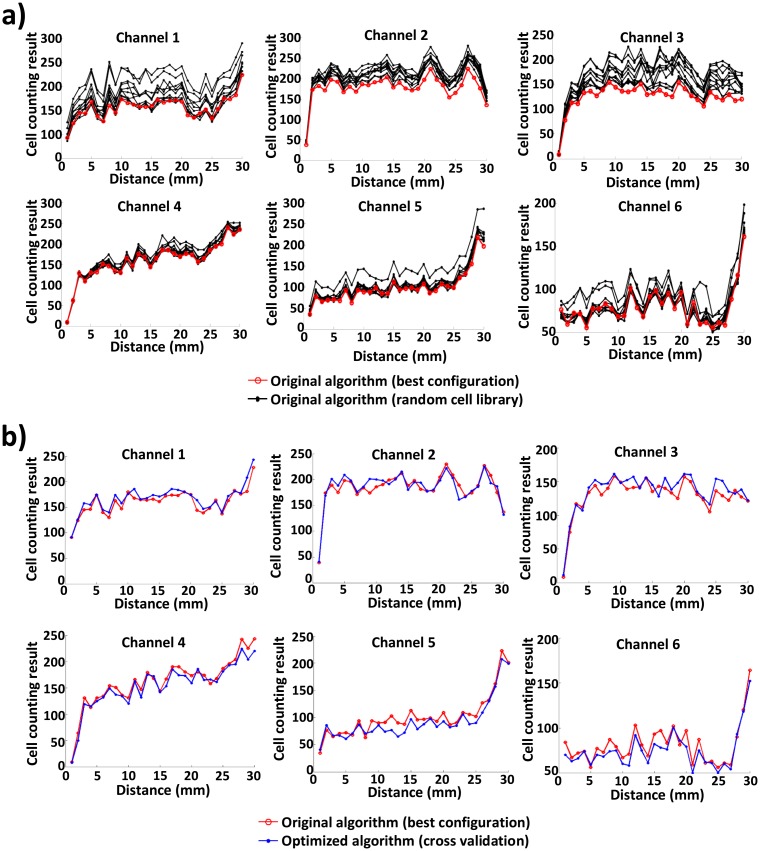
Evaluation of the counting accuracy of the original and optimized algorithms. **(a)** For each of six channels, 10 count results of the original algorithm, each with its own randomly-selected cell image library, are plotted based on the distance (up to 30 mm) from the inlet port of each channel; the count result of the original algorithm that yields the best match with manual counting is plotted, as well. In the original algorithm (using 150 random cell images), the number of counted cells varies a lot (up to 30%), due to the random generation of cell image library for each execution. **(b)** The counting results of the original algorithm (best configuration) and the optimized algorithm (via cross-validation) are compared. For each of the channels, a specific cell image library for the optimized algorithm is generated by using the rest of five channel images to conduct cross-validation. The optimized algorithm exhibits an average difference of 5.05%. This demonstrates the robustness and effectiveness of the proposed approach.

To compare the accuracy and runtime of the original algorithm with those of the optimized algorithm, we run the two algorithms 10 times for six channels of the blood sample image. Figure 6 shows the counting accuracy or the number of counted cells of the original and optimized algorithms. In Figure 6a, for each of six channels, 10 count results of the original algorithm, each with its own randomly-selected cell image library, are plotted based on the distance (up to 30 mm) from the inlet port of each channel; the count result of the original algorithm that yields the best match with manual counting [2] is plotted, as well. Likewise, the counting results of the original algorithm (best configuration) and the optimized algorithm (via cross-validation) are compared in Figure 6b. In the original algorithm (using 150 random cell images), the number of counted cells varies a lot (up to 30%), due to the random generation of the cell image library for each execution. By contrast, the number of counted cells in the optimized algorithm is very close to that of manual counting. The optimized algorithm exhibits an average difference of 5.05%. This demonstrates the robustness and effectiveness of the proposed approach.

**Figure 7.**
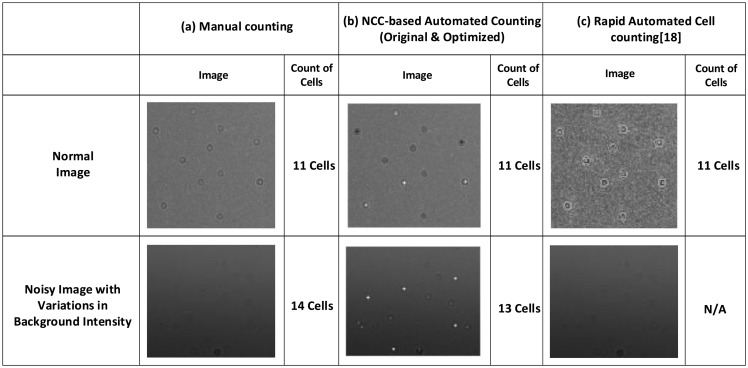
Comparison with manual counting and another cell counting algorithm [18]. With normal blood sample images, all three methods have the same results. For noisy images with intensity variations, NCC-based algorithms (both original and optimized) produce counting results close to those of manual counting, while [18] fails to reliably count cells.

Figure 7 compares NCC-based algorithms (both original and optimized) with manual counting and another cell counting algorithm [18] under two different scenarios. When normal blood sample images are applied, all three methods have the same counting results. By contrast, when the blood sample image is noisy and has variations in background variations, NCC-based algorithms produce counting results that are close to those of manual counting, while [18] fails to reliably count cells. This clearly shows that the proposed algorithm reliably deals with image anomalies, e.g., variations in background intensity, brightness and noise.

**Figure 8.**
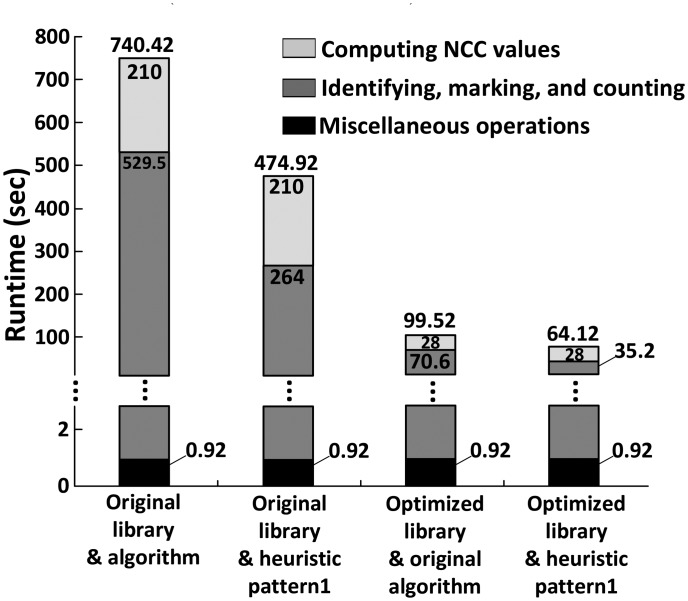
The runtime of the original algorithm and the optimized algorithms employing either or both of an optimized cell library to minimize the number of cell images and heuristic Pattern 1 to skip NCC computations for some points. The runtime of the original algorithm that evaluates NCC values for each and every point is, on average, 740.42 s, while that of the optimized algorithm is only 64.12 s, achieving almost a 11.5 × runtime reduction with accuracy loss less than 1%. The result also demonstrates that the cell library optimization reduces the runtime by 86.6% (from 740.42 s to 99.52 s), while heuristic Pattern 1 contributes to a 50.1% runtime reduction (from 529.5 s to 264 s).

Figure 8 shows the runtime of the original algorithm and three optimized algorithms employing either or both optimized cell libraries to minimize the number of cell images and heuristic Pattern 1 to skip NCC evaluations for some points, where the latter includes: (i) the optimized algorithm using NCC approximation with heuristic Pattern 1 only; (ii) the optimized algorithm using cell library optimization only; and (iii) the optimized algorithm employing two synergistic techniques together. The runtime of the original algorithm that evaluates NCC values for each and every point is, on average, 740.42 s, while that of the optimized algorithm is only 64.12 s, achieving almost an 11.5 × runtime reduction with accuracy loss less than 1%. The result also demonstrates that the cell library optimization reduces the runtime by 86.6% (from 740.42 s to 99.52 s), while heuristic Pattern 1 contributes to a 50.1% runtime reduction (from 529.5 s to 264 s).

## 4. Conclusions

Motivated by the need for fast and energy-efficient cell counting for mobile POC testing platforms, we proposed two synergistic techniques that optimize the cell image library and approximate the NCC-based cell counting algorithm and demonstrated their efficacy using an Android™ smart phone. First, optimizing the cell image library systematically eliminated duplicate and similar cell images that were manually chosen during the library creation process, reducing the runtime by nearly 87%. Second, approximating NCC evaluations by systematically skipping some points in a blood sample image also decreased the runtime by 50%. Note that a naive application of these two optimization techniques incurred some loss of counting accuracy. Thus, we developed a model for quantifying the loss of accuracy associated with these optimization techniques, and we demonstrated that such accuracy loss could be compensated for by adjusting the threshold value within an acceptable range (*i.e.*, >0.6). An Android™ smart phone running the original algorithm with the original cell image library containing 150 cells took 740.42 s to count all of the cells in a 3334 × 445 blood sample image. In contrast, the same Android™ smart phone running the optimized algorithm took 64.12 s with accuracy loss less than 1%, reducing the runtime by 11.5×.

## Figures and Tables

**Table 1.** Four heuristic patterns for NCC approximation, judiciously chosen to maximize the detection probability for a cell, as well as to reduce the runtime as much as possible. Employing Patterns 1, 2 and 3 reduces the runtime for evaluating NCC values by 50%∼75% at the expense of some accuracy loss (1.4%∼6.1%). Patterns 1 and 2 yield the highest detection probability (97.1%∼98.6%) and maintain the same amount of runtime reduction (50%). Pattern 3 achieves further reduction in runtime (75%) compared to Patterns 1 and 2, but its detection probability becomes lower (93.9%). Pattern 4 has the poorest detection probability (50.1%) and, hence, may not meet the accuracy requirement.

Description	Pattern	Runtime Reduction	Skipped Points	Total Points	Detection Probability
Heuristic Pattern 1	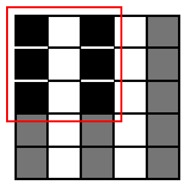	50%	3	9	98.6%
Heuristic Pattern 2	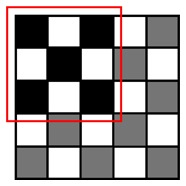	50%	4	9	97.1%
Heuristic Pattern 3	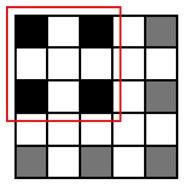	75%	5	9	93.9%
Heuristic Pattern 4	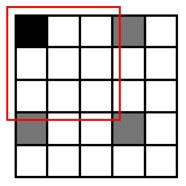	89%	8	9	50.1%
